# P-387. Exploring the Association Between Penicillin Allergy Labels (PAL), β-Lactam Utilization and Surgical Site Infections

**DOI:** 10.1093/ofid/ofae631.588

**Published:** 2025-01-29

**Authors:** Michael S Boger, Lisa Davidson, Anupama Neelakanta, Rupal K Jaffa, Catherine Passaretti

**Affiliations:** Atrium Health, Charlotte, North Carolina; Atrium Health, Charlotte, North Carolina; Atrium Health, Charlotte, North Carolina; Atrium Health, Charlotte, North Carolina; Advocate Health, Charlotte, North Carolina

## Abstract

**Background:**

Several studies have reported higher surgical site infection (SSI) risk in patients with a penicillin allergy label (PAL). We explore the association between PAL, receipt of β Lactam Antibiotics and SSI in patients undergoing hip and knee arthroplasty.

**Figure 1:**
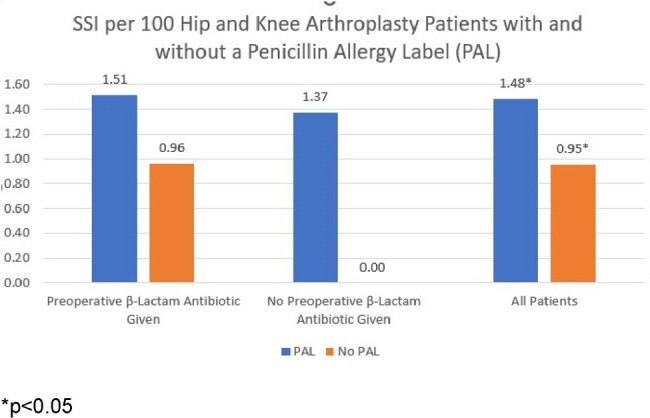
: SSI per 100 Hip and Knee Arthroplasty Patients with and without a Penicillin Allergy Label (PAL) Stratified by Receipt of Preoperative β-Lactam Antibiotic

**Methods:**

We performed a retrospective cohort study of hip (HPRO) and knee arthroplasty (KPRO) patients who underwent procedures in a large health system between August 1, 2022 and December 31, 2023 after implementation of a penicillin allergy risk assessment tool in the electronic health record. We compared demographic, clinical, procedural and SSI outcome data in patients with and without a PAL. Univariate and multivariable logistic regression were performed to assess for factors associated with risk of SSI.

**Table 1:**
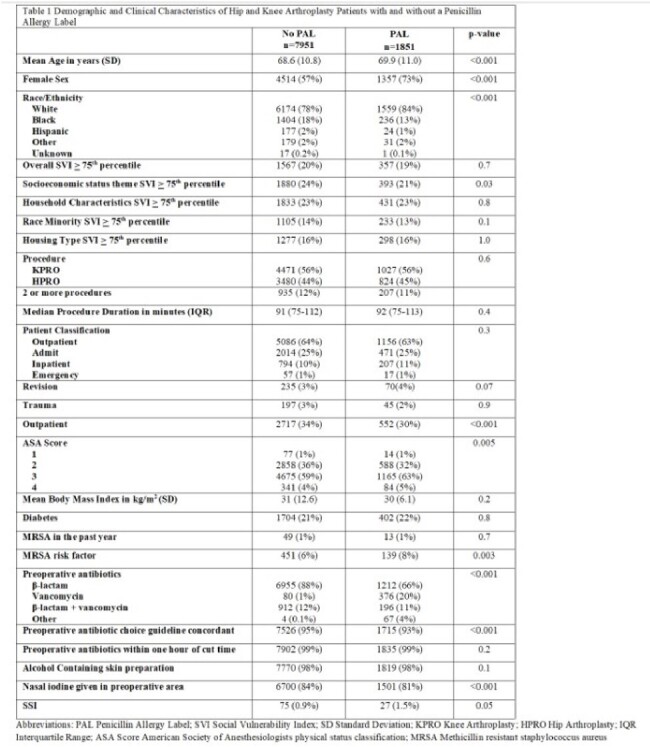
Demographic and Clinical Characteristics of Hip and Knee Arthroplasty Patients with and without a Penicillin Allergy Label

**Results:**

Of 9,802 patients who underwent 11,019 HPRO and KPRO procedures, 1851 (19%) reported a penicillin allergy and 102 (1.0%) had one or more SSI. PAL was more common in patients who were older, white, female, lived in areas with socioeconomic status Social Vulnerability Index (SVI) < 75th percentile and who had a higher American Society of Anesthesiologists (ASA) risk score. PAL patients were less likely to receive nasal iodine in the preoperative area. (Table 1) 77% of PAL patients received a preoperative β-lactam for prophylaxis with no reported adverse events. Patients with a PAL were 1.6 times more likely to have a SSI than those without a PAL. The trend toward higher risk of SSI in patients with PAL persisted regardless of whether the patient received preoperative β-lactam antibiotics (Figure 1). In the univariate analysis, day of surgery nasal iodine in the preoperative area and undergoing an outpatient procedure were protective whereas having a HPRO, revision, longer procedure, and higher ASA score were associated with higher odds of SSI. After adjustment for patient age, procedure type, revision, procedure duration, ASA score, outpatient procedure, receipt of a preoperative β-lactam antibiotic and receipt of nasal iodine in the preoperative area, PAL remained associated with 1.6 times higher odds of SSI (p=0.05).

**Table 2:**
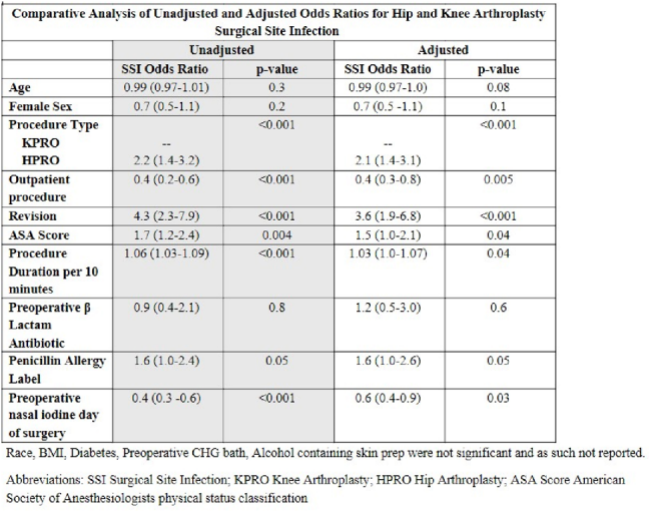
Comparative Analysis of Unadjusted and Adjusted Odds Ratios for Hip and Knee Arthroplasty Surgical Site Infection

**Conclusion:**

The increased risk of SSI in patients with PAL may not be fully explained by the lack of preoperative β-lactam antibiotic. More study is needed to assess further.

**Disclosures:**

**All Authors**: No reported disclosures

